# Theranostics in Hematological Malignancies: Cutting-Edge Advances in Diagnosis and Targeted Therapy

**DOI:** 10.3390/cancers17071247

**Published:** 2025-04-07

**Authors:** Bojana Bogdanovic, Florent Hugonnet, Christopher Montemagno

**Affiliations:** 1 Laboratory of Bioclinical Radiopharmaceutics, University Grenoble Alpes, INSERM, CHU Grenoble Alpes, 38000 Grenoble, France; bojana.bogdanovic@univ-grenoble-alpes.fr; 2 Nuclear Medicine Department, Centre Hospitalier Princesse Grace, 98000 Monaco, Monaco; florent.hugonnet@chpg.mc; 3 Biomedical Department, Centre Scientifique de Monaco, 98000 Monaco, Monaco

**Keywords:** hematologic malignancies, theranostics, alpha-emitters, personalized therapies, leukemia, lymphoma, myeloma, targeted therapy, cancer imaging, therapeutic strategies

## Abstract

Hematologic malignancies, including leukemia, lymphoma, and multiple myeloma, continue to challenge clinicians with complex treatment regimens that often involve significant side effects and limited success, especially in advanced stages. Recent advancements in nuclear medicine have introduced theranostic strategies that merge diagnostic imaging with targeted therapeutic approaches, offering the potential for more precise and personalized treatment. A key area of progress lies in the development of alpha-emitting radiopharmaceuticals, such as ^225^Ac, ^211^At, or ^212^Pb, which can deliver potent radiation directly to tumor cells, sparing healthy tissue and minimizing collateral damage. In parallel with these therapeutic advancements, molecular imaging using radiolabeled agents enables better disease monitoring, assessment of treatment efficacy, and personalized management of patients with hematologic malignancies. The integration of diagnostic imaging with radiotherapy allows for a more tailored approach, where treatment can be adjusted based on real-time information about tumor progression and response. This review examines the recent strides made in both the development of radiopharmaceuticals and their applications in molecular imaging, with a focus on the potential to improve precision, reduce toxicity, and optimize patient outcomes. The synergy between targeted therapy and molecular imaging represents a transformative shift in the management of hematologic malignancies. As these technologies evolve, they are poised to redefine treatment paradigms, offering new hope for patients and potentially improving survival rates with more effective and less toxic treatment options.

## Simple Summary

Hematologic malignancies, such as leukemia, lymphoma, and myeloma, are serious conditions that affect the blood, bone marrow, and lymphatic system. While current treatments can be effective, they often come with significant side effects or limited success. This review focuses on innovative theranostic approaches, which combine diagnosis and treatment into a single process. A promising area of development is the use of new agents, particularly those utilizing alpha-emitting particles, which can more precisely target cancer cells and minimize damage to healthy tissue. These advances aim to improve treatment precision, improve patient outcomes, and open new possibilities for personalized therapies. The insights from this review could help shape the future of treatments in nuclear medicine for hematologic malignancies and guide researchers and clinicians toward more effective therapeutic strategies.

## 1. Introduction

Hematological malignancies represent a highly heterogeneous group of cancers originating from the blood, bone marrow, and lymphatic system. These malignancies, encompassing leukemias, lymphomas, and multiple myeloma (MM), exhibit distinct genetic, molecular, and clinical characteristics that necessitate precise diagnostic and therapeutic strategies [1,2].

Leukemias arise from aberrant hematopoietic progenitors, leading to uncontrolled proliferation of dysfunctional leukocytes [3]. They are further classified into acute and chronic subtypes, with acute leukemias (acute myeloid leukemia [AML], acute lymphoblastic leukemia [ALL]) exhibiting aggressive progression, whereas chronic leukemias (chronic myeloid leukemia [CML], chronic lymphocytic leukemia [CLL]) follow a more indolent course [4]. Lymphomas, originating from lymphoid tissues, are broadly categorized into Hodgkin lymphoma (HL) and non-Hodgkin lymphoma (NHL), with NHL presenting a particularly diverse spectrum of molecular subtypes, ranging from indolent to highly aggressive forms [5]. MM, a malignancy of plasma cells, is marked by excessive monoclonal immunoglobulin production, leading to organ dysfunction, osteolytic lesions, and high relapse rates despite therapeutic advancements [6].

Conventional treatments for hematological malignancies typically include chemotherapy, radiation therapy, hematopoietic stem cell transplantation, and targeted therapies. Chemotherapy remains a cornerstone of treatment for many types of leukemia and lymphoma, while radiation therapy is frequently employed for localized disease in both hematological cancers and MM. Targeted therapies, such as tyrosine kinase inhibitors (e.g., imatinib for CML) and monoclonal antibodies (e.g., rituximab for NHL), have significantly improved outcomes by targeting specific molecular pathways involved in tumor progression [7]. However, these treatments often come with limitations, including non-specific toxicity, relapse due to resistance mechanisms, and the inability to effectively target minimal residual disease. Despite advances in these conventional therapies, the need for more personalized, effective, and less toxic treatment approaches remains a major challenge.

In this context, theranostics—an integrated approach combining molecular imaging and targeted radionuclide therapy (TRT)—offers a promising solution to overcome some of the limitations of conventional treatments. Theranostic strategies have already revolutionized the treatment of solid tumors, such as prostate cancer and neuroendocrine neoplasms, by enabling precise imaging-guided treatment delivery and minimizing off-target effects [8,9]. In hematologic malignancies, theranostics is gaining momentum, offering the potential to personalize treatment regimens and improve outcomes by targeting cancerous cells with high specificity while sparing healthy tissues. A notable advantage of theranostics over traditional therapies lies in its dual capability for diagnosis and therapy. By integrating molecular imaging techniques with radionuclide therapy, theranostic approaches enable real-time monitoring of disease progression, personalized treatment planning, and targeted drug delivery, all while minimizing systemic toxicity [10,11]. The ability to visualize tumor localization with agents such as positron emission tomography (PET) or single-photon emission computed tomography (SPECT) allows for more accurate patient stratification and treatment tailoring. Additionally, the use of radiolabeled antibodies or small molecules can directly deliver radiation to tumor cells, thus enhancing therapeutic efficacy while reducing collateral damage to surrounding healthy tissues.

**Figure 1:**
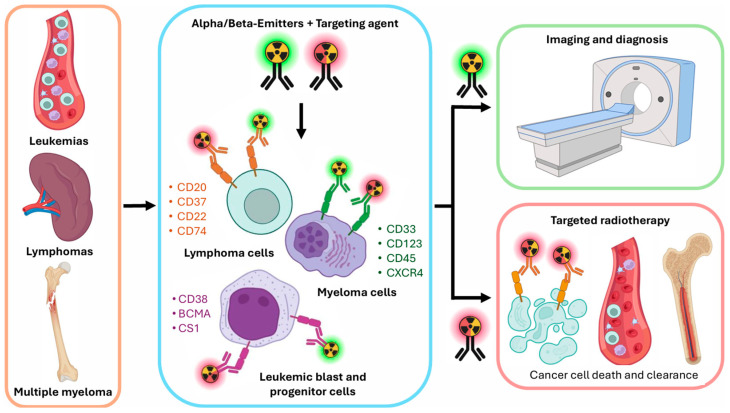
The figure illustrates the various types of malignant cells associated with hematological cancers, such as leukemias, lymphomas, and multiple myeloma (MM). Additionally, it highlights the potential surface targets expressed on these cells, which may be exploited for nuclear imaging and radioligand therapy using targeting agents labeled with alpha or beta emitters.

The early 2000s saw the clinical validation of radioligand therapy, culminating in the FDA approval of Yttrium-90 (^90^Y)-ibritumomab tiuxetan (Zevalin^®^) for follicular and B-cell non-Hodgkin lymphoma, as well as Iodine-131 (^131^I)-tositumomab (Bexxar^®^) for follicular lymphoma [12,13]. More recently, advancements in molecular radiopharmaceuticals and preclinical studies have unveiled novel theranostic targets, laying the foundation for future clinical applications. The most recent developments in the field are presented in Figure 1.

This review aims to provide a comprehensive exploration of the latest breakthroughs in theranostic approaches for hematological malignancies, highlighting emerging molecular targets, innovative radiopharmaceuticals, and their translational potential in improving patient outcomes. By integrating molecular diagnostics with TRT, theranostics offers a paradigm shift toward precision medicine in hemato-oncology, addressing the urgent need for more effective, personalized, and minimally invasive treatment strategies.

## 2. Principles of Nuclear Medicine in Hematological Malignancies

Theranostics is transforming oncology by integrating molecular imaging for diagnosis with radionuclide therapy for treatment, enabling personalized, real-time therapeutic monitoring and adaptive interventions [8,11]. In hematological malignancies, theranostics is particularly promising due to the high expression of disease-specific antigens (e.g., Cluster of Differentiation (CD)20, CD38, CD45, CD123) that can serve as molecular targets for radiopharmaceuticals. Early success in hemato-oncology theranostics was demonstrated with RIT, notably ^90^Y-ibritumomab tiuxetan and ^131^I-tositumomab, targeting CD20 in NHL [14,15,16]. However, their clinical adoption declined due to logistical challenges and the rise in alternative immunotherapies, such as chimeric antigen receptor (CAR) T cells and bispecific antibodies [17,18]. These innovations hold promise for overcoming therapeutic resistance, improving minimal residual disease detection, and expanding treatment options for patients with relapsed or refractory hematologic cancers.

### 2.1. Imaging Modalities in Theranostics for Hematological Malignancies

Molecular imaging plays a crucial role in theranostics by enabling precise detection, staging, and real-time therapeutic monitoring of hematological malignancies. PET and SPECT are the primary imaging techniques used to assess radiopharmaceutical distribution and tumor response. PET imaging, particularly with tracers like ^68^Ga- and ^18^F-labeled agents, offers high sensitivity and specificity for detecting malignant cells expressing molecular targets such as CD20, CD38, or CXCR4 [19,20]. PET-based radiotracers allow for non-invasive whole-body tumor burden assessment, guiding personalized treatment planning. SPECT, often coupled with ^111^In- or ^99m^Tc-labeled tracers, provides functional imaging with high specificity but lower sensitivity compared to PET [21]. It remains valuable for monitoring radiolabeled antibody therapies and assessing bone marrow infiltration. PET imaging has demonstrated superior sensitivity in detecting minimal residual disease (MRD) and treatment response, whereas SPECT is frequently utilized for dosimetry and treatment monitoring in radionuclide therapy. Clinical trials evaluating novel PET tracers, such as ^68^Ga-pentixafor for CXCR4-expressing malignancies, underscore the evolving role of advanced molecular imaging in hematology.

### 2.2. Types of Radioactive Emitters Used in the Treatment of Hematological Malignancies

#### 2.2.1. Beta(β)-Emitters

β-emitting radionuclides are widely used in nuclear medicine due to their ability to penetrate millimeter-range distances in tissues, making them suitable for targeting hematologic malignancies while sparing surrounding normal structures. With a half-life of 6.7 days and a moderate β energy (average penetration range ~7 mm), Lutetium-177 (^177^Lu) is ideal for delivering controlled radiation doses to tumor cells [22]. It has been extensively studied in neuroendocrine tumors and prostate cancer, but recent research suggests its potential in CD38-targeted therapy for hematological malignancies [23]. Besides ^177^Lu, ^90^Y, a high-energy pure β-emitter with a shorter penetration range (~5 mm), has been successfully used in ^90^Y-ibritumomab tiuxetan for B-cell lymphomas, leading to the approval by the FDA [24]. In terms of clinical applications, β-emitters are effective for treating tumors of a larger size, where the radiation can penetrate and deliver cytotoxic effects. The sensitivity of β-emitters makes them suitable for localized tumor control, but their range limits effectiveness for isolated tumor cells or micrometastases. As a result, β-emitters are best suited for targeting established tumors but might be less effective at controlling minimal residual disease [25]. Moreover, despite their ability to provide targeted therapy, β-emitters still carry the risk of off-target toxicity, as the radiation can impact nearby healthy tissues within the penetration range.

#### 2.2.2. Alpha(α)-Emitters

α-emitters represent the next frontier in targeted radiotherapy, offering high linear energy transfer (LET) and short tissue penetration (~50–100 µm), which results in high cytotoxicity with minimal off-target effects. With a half-life of 10 days, Actinium-225 (^225^Ac): emits high-energy α-particles, demonstrating superior tumoricidal effects compared to β-emitters [26,27]. Some recent examples include tracers labeled with ^225^Ac, Astatine-211 (^211^At) or Lead-212 (^212^Pb) [28]. For instance, studies on ^225^Ac-labeled CD38-targeting agents in MM have shown encouraging results in resistant cases [29]. As a β-to- α generator, ^212^Pb is gaining attention for its theranostic potential, particularly in CD22- and CD37-targeted radiopharmaceuticals for NHL [30,31,32]. Due to its short half-life (7.2 h) and high LET, ^211^At is also being explored in leukemia therapy, where its limited range could be advantageous in targeting bone marrow-residing tumor cells. α-emitters are highly effective for targeting small clusters of tumor cells and even single cancerous cells due to their potent cytotoxicity. Their short tissue penetration ensures minimal exposure to surrounding healthy tissues, which is a key advantage when targeting bone marrow-residing tumor cells or minimal residual disease. Clinical data from studies using ^225^Ac-labeled CD38-targeting agents in multiple myeloma have shown promising results, particularly in cases resistant to other treatments. Additionally, ^212^Pb, a β-to-α generator, is being explored for its potential in treating NHL, especially with its ability to target CD22- and CD37-expressing tumor cells. Despite their advantages, α-emitters face challenges related to production and availability. The complex production processes and limited supply of isotopes like ^225^Ac and ^212^Pb hinder their widespread clinical use. Furthermore, the short half-life of α-emitters poses logistical challenges in ensuring timely administration. Nonetheless, their high efficacy and specificity make them a promising avenue for advancing theranostic treatments in hematologic malignancies.

#### 2.2.3. Auger-Emitters

With a half-life of 6.96 days, an emission spectrum that includes β− particles (Eβ−, av = 154 keV), γ-rays at 48.9 keV and 74.6 keV, which are well-suited for SPECT imaging, and Auger electrons, 161-terbium (^161^Tb) emerges as a promising radionuclide for theranostic applications. The significant emission of electrons with energies below 40 keV holds substantial potential for targeting micrometastases [33]. To date, there are no studies involving this radionuclide in hematology.

Despite their potential, Auger emitters have yet to see widespread clinical application in hematology, and there are currently no studies specifically involving this radionuclide in hematologic cancers [34]. The key limitation of Auger emitters lies in the need for highly sensitive imaging and dosimetry techniques to assess their effectiveness, given their low-energy emissions. Furthermore, while their potential for targeting micrometastases is substantial, clinical validation is needed to determine their role in the treatment of hematological malignancies.

#### 2.2.4. Comparing β-Emitters and α-Emitters

**Figure 2:**
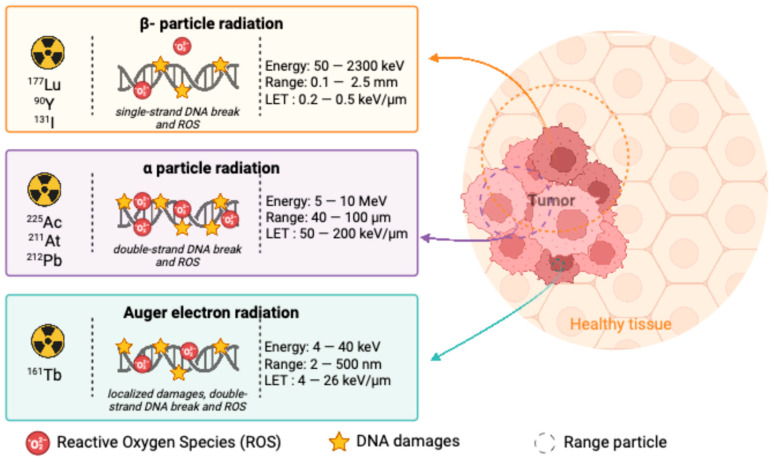
The different types of radioactive emitters used in the treatment of hematological malignancies, including β-emitters, α-emitters, and Auger electrons. The figure provides a comparative overview of β-emitters, α-emitters, and Auger electrons, highlighting their respective penetration ranges, linear energy transfer (LET), and mechanisms of action. β-emitters, such as ^177^Lu and ^90^Y, deliver moderate energy over a longer range, making them suitable for bulky tumors. α-emitters, including ^225^Ac, ^211^At, and ^212^Pb, offer high LET with short penetration, enabling highly localized cytotoxicity ideal for targeting isolated malignant cells. Auger electrons, characterized by extremely short-range deposition, require precise targeting of the nucleus for maximal DNA damage, offering a unique approach to molecular radiotherapy.

In terms of efficacy, α-emitters offer superior tumoricidal effects due to their high LET, making them more effective for treating small or isolated tumor cells, minimal residual disease, and resistant tumor populations. The high toxicity of α-particles can overcome some of the challenges posed by tumor heterogeneity and resistance mechanisms that β-emitters may not address. However, β-emitters like ^177^Lu and ^90^Y remain valuable for treating larger tumors and offering broader tissue penetration. The safety profile of α-emitters is generally superior due to their localized tissue penetration, which minimizes off-target radiation exposure. In contrast, β-emitters, while offering deeper tissue penetration, may inadvertently affect surrounding healthy tissues within their range, leading to potential toxicity. Real-world clinical data further highlight these distinctions. β-emitters have been extensively studied and are FDA-approved for use in NHL and other hematological malignancies, whereas α-emitters are still in the experimental phase with promising preclinical results. More clinical studies are required to confirm the long-term benefits and safety of α-emitters in hematology. Figure 2 provides a comparative overview of the different types of radioactive emitters used in the treatment of hematological malignancies, illustrating their distinct physical properties, mechanisms of action, and therapeutic implications.

### 2.3. TRT to Overcome Resistance Mechanisms, Improve Selectivity and Reduce Toxicity

TRT presents a compelling alternative to conventional treatments by addressing key limitations associated with monoclonal antibodies (mAbs), chemotherapy, and CAR-T cell therapy in hematologic malignancies [35]. One major challenge with mAbs is antigen escape or downregulation, as observed in post-CAR-T relapses where CD19 expression is lost, rendering the therapy ineffective [36]. In contrast, TRT delivers cytotoxic radiation that can sensitize tumors to immune checkpoint blockade [37]. Unlike chemotherapy, which relies on cell cycle activity and is often ineffective against quiescent tumor cells, radionuclides induce DNA single or double-strand breaks irrespective of mitotic status, making them highly effective even against slow-dividing malignant cells [38]. While CAR-T therapy has revolutionized hematologic cancer treatment, it is frequently associated with cytokine release syndrome (CRS) and neurotoxicity, complications that can be severe or life-threatening [39]. In contrast, α-emitting radionuclides provide a more localized cytotoxic effect without systemic immune overactivation, offering a potentially safer therapeutic option or combination strategy. Additionally, TRT is characterized by minimal off-target effects, as the short penetration range of α-particles limits damage to surrounding healthy tissues, significantly reducing bone marrow suppression compared to chemotherapy [40]. However, organs that physiologically bind radiotracers, such as the parotid glands in PSMA-targeted therapy, may still be at risk for toxicity. These advantages position TRT as a powerful tool to overcome treatment resistance, enhance selectivity, and improve safety profiles, paving the way for next-generation precision medicine approaches in hematologic oncology.

## 3. Recent Advancements in Hematological Malignancies, from Preclinical to First in Human Studies

### 3.1. Targets in B-Cell Lymphomas

#### 3.1.1. CD20

CD20 is a well-established therapeutic target in B-cell lymphomas due to its high expression on malignant B cells and absence on hematopoietic stem cells, allowing selective targeting. mAbs like rituximab have revolutionized treatment, but resistance mechanisms, including CD20 downregulation and immune escape, necessitate alternative strategies. RIT has emerged as a potent approach to enhance CD20-directed therapies. ^90^Y-ibritumomab tiuxetan (Zevalin) and ^131^I-tositumomab were among the first radiolabeled anti-CD20 agents approved, demonstrating improved responses, especially in relapsed or refractory follicular lymphoma. However, their clinical adoption has been limited by concerns over myelosuppression and logistical challenges.

Recent advancements in next-generation radiopharmaceuticals, including ^177^Lu-labeled antibodies, aim to improve therapeutic efficacy while minimizing toxicity. Preclinical and early clinical studies suggest that ^177^Lu-based approaches may offer better tumor penetration, and a favorable safety profile compared to earlier RIT agents. Notably, ^177^Lu-DOTA-rituximab demonstrated promising biodistribution and dosimetry in patients with CD20+ lymphoma, supporting its potential for future clinical applications [41,42,43]. Other anti-CD20 mAbs, such as ofatumumab, have also shown significant anti-tumor efficacy in preclinical models, including the subcutaneous Raj mice model [44].

Beyond β-emitting radiopharmaceuticals, α-emitting therapies such as ^225^Ac-anti-CD20 are being investigated for their ability to induce potent cytotoxicity with minimal off-target effects. In an aggressive disseminated tumor model, ^225^Ac-ofatumumab demonstrated curative potential when administered eight days post-cell injection, highlighting its strong translational potential as a next-generation therapeutic [45]. Efforts to integrate PET imaging into CD20-targeted therapies have also advanced, enabling improved patient selection and response monitoring. Imaging with Zirconium-89 (^89^Zr)-rituximab or ofatumumab has been explored for evaluating treatment efficacy in NHL mouse models [46,47,48]. Additionally, Copper-64 (^64^Cu)-DOTA-rituximab PET/CT has been reported as a powerful tool for diagnosing and tracking treatment responses in NHL [49]. These advancements position CD20-targeted radiotherapy as a promising strategy in precision medicine for B-cell lymphomas, with ongoing studies seeking to refine dosing strategies and optimize patient selection.

#### 3.1.2. CD37

CD37 is a transmembrane protein highly expressed in mature B cells, making it a promising therapeutic target in B-cell malignancies, particularly NHL. Unlike CD20, CD37 plays a crucial role in cell survival and signal transduction, offering a distinct avenue for targeted therapies [50,51]. CD37-directed strategies include antibody-drug conjugates, radiolabeled antibodies, and theranostic approaches. One of the first advancements in CD37-targeted RIT was driven by lilotomab, a murine monoclonal antibody that paved the way for radiopharmaceutical development [52].

Following promising preclinical results obtained in mice model of NHL, lilotomab was initially used as a pre-targeting agent in combination with ^177^Lu-lilotomab satetraxetan (Betalutin^®^) to reduce off-target toxicity, particularly bone marrow suppression [53,54,55,56]. In a first-in-human study for relapsed indolent NHL, Betalutin^®^ showed promising efficacy, with tumor-absorbed dose (tTAD) ≥ 200 cGy correlating with an improved metabolic response on FDG PET/CT [57]. Moreover, its combination with rituximab demonstrated synergistic tumor suppression in NHL xenograft models, enhancing CD20 expression and potentially improving clinical outcomes with combined CD37- and CD20-targeted therapies [52]. However, the murine origin of Betalutin^®^ posed limitations for repeated administration due to immunogenicity concerns, necessitating next-generation alternatives [52,57].

To address these challenges, a fully humanized anti-CD37 antibody, NNV003, was developed with a higher affinity for CD37, aiming to enhance therapeutic efficacy while reducing immune responses. This antibody serves as the backbone for advanced theranostic applications, including ^177^Lu-NNV003, which demonstrated high tumor uptake and durable responses in preclinical models [52]. A companion PET imaging agent, ^89^Zr-N-sucDf-NNV003, was also developed to accurately predict CD37-targeting and biodistribution of ^177^Lu-NNV003 RIT, supporting its role as a noninvasive tool for patient selection and dose optimization [58].

In parallel, α-emitting radiopharmaceuticals such as ^212^Pb-NNV003 are being explored to deliver high LET radiation directly to CD37-positive cells, potentially overcoming resistance mechanisms [59]. Additionally, novel in vitro studies suggest that combining CD37-TRT with PARP inhibitors could enhance therapeutic efficacy by increasing tumor radiosensitivity via DNA damage response inhibition [60]. With ongoing clinical trials and emerging theranostic applications, CD37-directed RIT is poised to reshape the treatment landscape for B-cell malignancies, offering a novel approach for patients resistant to conventional therapies.

#### 3.1.3. CD22

CD22 has emerged as a compelling therapeutic target for patients who have experienced treatment failure after CD19-targeting immunotherapies, including CD19-directed CAR T-cell therapy [61]. As a B-cell-specific sialic acid-binding immunoglobulin-like lectin (Siglec), CD22 plays a crucial role in B-cell receptor (BCR) signaling and endocytosis, making it an attractive target in hemato-oncology. It is highly expressed in various B-cell malignancies, including ALL, diffuse large B-cell lymphoma (DLBCL), and Burkitt’s lymphoma, particularly in cases where CD19 expression is lost or downregulated [62].

Several CD22-targeting strategies have been developed, with promising clinical outcomes. Inotuzumab ozogamicin, an anti-CD22 antibody-drug conjugate, has demonstrated significant efficacy in relapsed/refractory B-cell ALL (B-ALL), leading to its FDA approval [63]. Beyond antibody-drug conjugates, CD22-directed RIT represents a powerful approach, particularly for patients with refractory disease. Preclinical studies have highlighted the potential of ^177^Lu-labeled CD22-specific radioimmunoconjugates, showing potent anti-tumor activity in NHL models [64]. Notably, a dual-targeting strategy combining ^177^Lu-based CD22-specific radioimmunoconjugates with rituximab has demonstrated high treatment efficacy in Burkitt’s lymphoma xenograft models, suggesting a synergistic effect between anti-CD22 and anti-CD20 therapies [65]. Additionally, ^211^At-labeled anti-CD22 RIT has emerged as a promising alternative for B-cell malignancies, benefiting from the short-range, high-energy α-particle emissions, which enhance tumor cell killing while minimizing off-target toxicity [66].

Despite these advancements, challenges remain, including heterogeneous CD22 expression, potential antigen escape, and treatment resistance. Future directions include optimizing combinations with other immunotherapies, such as CD19/CD22 bispecific CAR-T cells or dual-antigen targeting radioimmunoconjugates, to enhance the durability of response and overcome resistance mechanisms [67]. Given the increasing evidence supporting the efficacy of CD22-directed strategies, this antigen remains a promising target for improving outcomes in patients with B-cell malignancies.

#### 3.1.4. CD74

CD74 has emerged as a promising therapeutic target in B-cell malignancies due to its high expression in aggressive lymphomas, including DLBCL, MCL, CLL, and MM. Beyond its role in antigen presentation as the invariant chain (Ii) of MHC class II, CD74 also acts as a signaling receptor for macrophage migration inhibitory factor (MIF), promoting cell survival and proliferation [68].

Several therapeutic approaches targeting CD74 are under investigation. Milatuzumab, a humanized anti-CD74 monoclonal antibody, has demonstrated preclinical efficacy by inducing direct apoptosis and enhancing antibody-dependent cellular cytotoxicity (ADCC) [69]. Additionally, antibody-drug conjugates (ADCs) such as STRO-001 have shown promise in preclinical models of hematologic malignancies, offering targeted cytotoxic delivery with reduced off-target effects [70,71].

CD74-directed RIT has also been explored as a therapeutic strategy. A study evaluating a Bismuth-213 (^213^Bi)-labeled anti-CD74 antibody demonstrated potential efficacy against B-cell lymphoma [72]. However, despite encouraging preclinical findings, clinical development in this area has been limited, and no clinical data are currently available.

### 3.2. Targets in Leukemias (Myeloid and Lymphoid)

#### 3.2.1. CD33

CD33 is a myeloid differentiation antigen highly expressed in AML and has become a key therapeutic target in the treatment of this disease. Given its restricted expression on myeloid progenitors and leukemic blasts, CD33-directed therapies have been developed to improve AML outcomes while minimizing off-target toxicity. Several therapeutic approaches have been explored, including mAbs, ADCs such as gemtuzumab ozogamicin (Mylotarg^®^, approved by the FDA), and RIT [73,74,75]. Lintuzumab, an anti-CD33 monoclonal antibody, has been radiolabeled with various isotopes for both imaging and therapeutic applications. ^89^Zr-labeled lintuzumab has demonstrated effective tumor targeting in OCI-AML3 xenograft models, highlighting its potential for PET imaging in AML [76]. Similarly, Technetium-99m (^99m^Tc)-labeled nanobody (Nb) has been evaluated in THP-1 tumor-bearing mice, providing a promising tool for CD33-targeted imaging [77].

CD33-directed RIT has also shown encouraging results, highlighting a potential to be evaluated in large-scale clinical trials. For instance, Thorium-227 (^227^Th)-labeled CD33 antibodies have exhibited potent anti-tumor effects in HL-60 xenograft mouse models [78,79]. Moreover, ^225^Ac-labeled lintuzumab has demonstrated significant therapeutic efficacy in preclinical AML models [80], with further validation in AML patients [81]. Indeed, therapy using ^225^Ac-lintuzumab was reported to be feasible with an acceptable safety profile. Elimination of circulating blasts or reductions in marrow blasts were observed across all dose levels. These findings underscore the potential of CD33-targeted RIT as an emerging approach for AML treatment.

#### 3.2.2. CD123

CD123, the α-subunit of the interleukin-3 receptor (IL-3Rα), is overexpressed in AML and other hematologic malignancies, making it an attractive therapeutic target. Its restricted expression on normal hematopoietic stem cells and high prevalence on leukemic stem cells (LSCs) suggest that CD123-targeted therapies could improve treatment specificity while sparing normal hematopoiesis [82].

Several therapeutic strategies have been developed, including mAbs, ADCs, and CAR-T cell therapies [83]. Among them, tagraxofusp (SL-401), a CD123-targeting fusion protein conjugated to diphtheria toxin, has been FDA-approved for blastic plasmacytoid dendritic cell neoplasm (BPDCN) and is being evaluated in AML [84].

IMGN632, an anti-CD123 ADC, has also shown promising clinical activity in AML and BPDCN [85,86]. Additionally, CD123-targeted CAR-T cell therapies are undergoing clinical evaluation, with some demonstrating early signs of efficacy [85].

CD123-directed RIT has shown promising potential. In preclinical studies, a single dose of ^211^At-labeled mAbs targeting CD123 (1.48 MBq) decreased tumor burden and significantly prolonged survival in MOLM-13 AML xenograft models [87]. These findings were the first to show the potential of α-particle-based RIT for eradicating CD123-positive leukemic cells while minimizing damage to surrounding tissues.

Further investigations into CD123-directed RIT, particularly in combination with other targeted therapies, could pave the way for improved treatment outcomes in AML patients, especially those with refractory or relapsed disease.

#### 3.2.3. CD45

CD45 is a transmembrane protein tyrosine phosphatase expressed on all hematopoietic cells except mature erythrocytes [88]. Given its broad expression across leukemic cells, CD45-directed RIT has been explored to enhance myeloablative conditioning, improving transplantation outcomes while sparing non-hematopoietic tissues. This strategy is particularly promising in AML and other hematologic malignancies.

Among the most studied approaches, ^131^I-apamistamab, an anti-CD45 monoclonal antibody, has demonstrated significant potential in both preclinical and clinical settings. The Phase III SIERRA trial showed that ^131^I-apamistamab-based conditioning led to a higher deep complete remission (dCR) rate compared to conventional care in older patients with relapsed/refractory (R/R) AML [89]. Moreover, the treatment was well tolerated, addressing a critical unmet clinical need in this high-risk population.

Beyond ^131^I-apamistamab, other CD45-targeted radiopharmaceuticals have been explored. ^90^Y-DOTA-BC8 was evaluated in a Phase I trial for MM, demonstrating potential as a myeloablative therapy [90]. Another Phase I study investigated dose escalation of ^131^I-labeled CD45 antibodies, further validating the feasibility of this approach [91].

These findings highlight CD45-directed RIT as a promising strategy for improving conditioning regimens before hematopoietic stem cell transplantation (HSCT) and for targeting resistant leukemic cells in AML and other blood cancers. Further studies should focus on optimizing dosing, reducing toxicity, and exploring combination strategies to enhance efficacy.

#### 3.2.4. CXCR4

CXCR4, a chemokine receptor involved in cell homing and migration, plays a pivotal role in leukemia cell survival, proliferation, and therapy resistance. Its overexpression in various hematologic malignancies, particularly AML and MM, has established CXCR4 as a compelling therapeutic target [92,93]. By mediating interactions between leukemic cells and the bone marrow microenvironment, CXCR4 facilitates tumor cell retention and contributes to drug resistance and disease progression [94].

Radiopharmaceutical strategies targeting CXCR4 have been developed to disrupt these protective niches and improve treatment efficacy. Among the most studied agents, ^177^Lu-pentixather and ^90^Y-pentixather—radiolabeled analogs of the CXCR4 antagonist pentixafor—have shown promise in both imaging and therapeutic applications [95,96]. Preclinical and early clinical studies have demonstrated their capacity for effective tumor targeting, bone marrow ablation, and potential eradication of minimal residual disease in hematologic cancers [97,98]. Expanding the potential of CXCR4 targeting beyond hematologic malignancies, recent studies have also explored the use of ^212^Pb/^203^Pb-labeled agents in CXCR4-expressing tumors, demonstrating therapeutic efficacy in murine models of small cell lung cancer [99]. While early results are promising, ongoing studies aim to optimize dosing strategies, improve safety profiles, and identify patient populations that could derive the greatest benefit from CXCR4-targeted RIT.

### 3.3. Targets in Multiple Myeloma

#### 3.3.1. CD38

CD38 is a multifunctional transmembrane glycoprotein highly expressed on plasma cells and overexpressed in MM, making it a key therapeutic and diagnostic target. Its role in cell adhesion, signal transduction, and calcium signaling further underscores its importance in MM pathophysiology [100]. The success of CD38-targeting mAbs, such as daratumumab and isatuximab, has revolutionized MM treatment [101,102,103]. However, challenges such as resistance and minimal residual disease (MRD) persistence necessitate novel strategies, including radiopharmaceutical approaches. Several radiolabeled antibodies and nanobodies targeting CD38 have been explored for both imaging and therapeutic applications.

mAbs such as daratumumab and isatuximab have been radiolabeled with ^89^Zr and evaluated in mouse models of MM. Specifically, ^89^Zr-DFO-isatuximab enabled the visualization of disseminated MM as well as Burkitt’s lymphoma models [104]. In clinical settings, ^89^Zr-daratumumab showed promising imaging capabilities in human MM, underscoring its potential as a diagnostic tool [105]. In contrast to these antibodies, which require longer imaging times, nanobodies have been developed more recently for this purpose. Examples include ^99m^Tc-CD3813, Fluor-18 (^18^F)-Nb1053, Gallium-68 (^68^Ga)-TOHP-CD3813, and ^68^Ga--NOTA-Nb1053, which have been validated in several MM mouse models [106,107,108,109]. Additionally, peptides like ^64^Cu-NODAGA-PEG4-SL022-GGS or ^68^Ga-AJ206 have been developed for imaging MM, demonstrating a high lesion-to-background ratio at early time points [110,111]. All these tracers have proven to be specific and effective for visualizing MM, thus providing a solid foundation for the clinical translation of such tracers for MM detection.

In terms of therapeutic applications, radiolabeled anti-CD38 antibodies have also shown promising results. Anti-CD38 single-domain antibodies (sdAbs) labeled with ^177^Lu demonstrated high tumor uptake in CD38⁺ MM xenografts, suggesting their potential in targeted therapy [112]. Additionally, ^212^Pb-daratumumab showed cytotoxic efficacy against RPMI8226 cells both in vitro and in vivo, reinforcing its therapeutic potential for MM treatment [113]. Similarly, ^213^Bi-anti-CD38 effectively targeted MM xenografts in preclinical models, offering further evidence for its therapeutic benefits [114]. The ^211^At-labeled anti-CD38 showed therapeutic advantages in a disseminated MM disease model [115], while daratumumab labeled with ^225^Ac or ^177^Lu was tested in a preclinical model of disseminated MM, with ^225^Ac demonstrating superior cytotoxicity [116]. These promising findings indicate that radiolabeled anti-CD38 antibodies could play a significant role in the treatment of MM, although further clinical studies are necessary to fully evaluate their therapeutic potential.

#### 3.3.2. B-Cell Maturation Antigen (BCMA)

BCMA, a member of the tumor necrosis factor receptor superfamily, is selectively expressed on plasma cells and overexpressed in MM, making it a highly attractive therapeutic target. Its limited expression on normal tissues reduces off-target effects, establishing BCMA as a central focus in the development of targeted therapies [117]. BCMA-targeted strategies have significantly advanced MM treatment, especially with the success of ADCs, bispecific T-cell engagers (BiTEs), and CAR-T cell therapies [118,119,120]. However, challenges remain in effectively stratifying patients who would most benefit from BCMA-targeted therapies and in real-time monitoring of therapeutic efficacy.

Recent developments in nuclear medicine have introduced novel theranostic approaches. Notably, ^89^Zr-DFO-BCMAh230430 and ^177^Lu-DTPA-BCMAh230430 have shown promise as dual-purpose agents for both imaging and radioligand therapy in BCMA-expressing MM, as demonstrated in preclinical murine models [121]. These agents enable precise tumor visualization and targeted radiotherapy, offering potential improvements in patient selection and treatment monitoring. Additionally, α-emitting therapies have emerged as potent strategies for MM treatment. Preclinical studies investigating ^211^At-BCMA-B10, a human IgG1 radiolabeled with ^211^At, demonstrated its ability to eradicate MM cells in murine models [122]. These findings underscore the potential of BCMA-targeted radioligand therapies to eliminate minimal residual disease and improve patient outcomes. Further clinical trials are warranted to evaluate safety, efficacy, and integration into existing treatment regimens for MM.

#### 3.3.3. Signaling Lymohocytic Activation Molecule Family Member 7 (SLAMF7; CS1)

CS1 is a surface glycoprotein predominantly expressed on plasma cells, particularly in MM, with limited expression in normal tissues [123]. Its involvement in immune modulation and cell adhesion makes CS1 a valuable therapeutic target. The mAbelotuzumab, which enhances natural killer (NK) cell-mediated cytotoxicity, has been FDA-approved for MM treatment, underscoring the clinical relevance of CS1 targeting [124]. In the field of theranostics, radiolabeled CS1-targeted agents have shown promising results. For diagnostic applications, ^89^Zr-labeled elotuzumab has been evaluated for PET imaging in MM.1S-CG cell-bearing mice, enabling precise tumor localization and offering a potential tool for disease monitoring and treatment response evaluation [125]. On the therapeutic side, ^225^Ac-labeled sdAb demonstrated potent cytotoxic effects in preclinical MM models. Interestingly, this treatment also increased CD8⁺ T-cell infiltration and PD-L1 expression, suggesting that CS1-directed radioligand therapy could be combined with immunotherapies to enhance anti-tumor responses [126]. These findings highlight the dual potential of CS1-targeted radiopharmaceuticals in both MM diagnosis and treatment. The integration of α-emitters like ^225^Ac with CS1-specific binders may open new avenues for eradicating minimal residual disease, while radiolabeled antibodies such as ^89^Zr-elotuzumab offer enhanced imaging capabilities. Future research should focus on optimizing these strategies to improve patient outcomes, reduce toxicity, and explore potential synergies with existing immunotherapies.

All the recent development in the field are summarized in Table 1.

### 3.4. Other Emerging Targets in Hematological Malignancies

Besides the well-established targets discussed in this review, other emerging biomarkers are being explored across various hematological malignancies. These novel targets open new avenues for both diagnostic and therapeutic strategies, broadening the scope of precision medicine in hematology.

#### 3.4.1. CD70

CD70, a member of the tumor necrosis factor (TNF) family, is transiently expressed on activated T and B cells under normal physiological conditions but is aberrantly overexpressed in various hematological malignancies, notably leukemias [128]. In contrast to CD123 and CD33, CD70 is not expressed on normal hematopoietic stem cells, suggesting that CD70-directed therapies could be effective without affecting hematopoiesis. While extensively studied in immunotherapy—such as ADCs, CAR-T cell therapies, and mAbs—CD70 remains relatively underexplored in the field of nuclear medicine [129]. However, its selective expression profile offers promising potential for both diagnostic imaging and TRT. In preclinical models, CD70-targeting radioligands have shown promise, including CD70-TTC, a novel thorium-227 (^227^Th) conjugate, which holds great potential for the treatment of CD70-expressing tumors [130]. While most research has focused on solid tumors such as renal cell carcinoma, the selective expression profile of CD70 in hematological malignancies suggests that CD70-directed radioligand therapies could be explored for leukemias and lymphomas. Ongoing investigations will be crucial to determine the feasibility of CD70-targeted theranostics in hematology.

#### 3.4.2. DOTA-TATE

In addition to established targets for MM, such as CD38 and CS1, recent research has explored alternative strategies, including the potential repositioning of DOTA-TATE as both a diagnostic and therapeutic tool in MM [131]. Traditionally employed in the management of neuroendocrine tumors for imaging and radionuclide therapy, DOTA-TATE targets somatostatin receptors (SSTR), particularly SSTR2, and is now under evaluation in MM (NCT04379817). Its expression in MM has prompted clinical evaluation, with studies suggesting that SSTR-targeted imaging may complement conventional approaches like ^18^F-FDG PET/CT. The SCARLET study (Somatostatin Receptors Imaging in Relapsing and Refractory Multiple Myeloma Patients) in investigating the feasibility of using ^68^Ga-DOTA-TATE PET/CT imaging to detect somatostatin receptor expression in relapsing and refractory MM patients. This study is particularly important as it aims to assess whether SSTR-targeted imaging can offer greater sensitivity than ^18^F-FDG PET/CT, especially in cases where FDG shows limited sensitivity. This could significantly improve the detection of myeloma lesions and enable more precise monitoring of disease progression. Beyond diagnostic imaging, the therapeutic potential of ^177^Lu-DOTA-TATE deserves further investigation in MM. By harnessing DOTA-TATE’s affinity for SSTR-expressing myeloma cells, radionuclide therapy could offer a novel approach to target resistant or residual disease, contributing to more personalized treatment strategies. Although still in its early stages, the repositioning of DOTA-TATE holds promise for enhancing both imaging precision and TRT in MM. Ongoing and future studies will be essential to validate its clinical efficacy and to clarify its role within the evolving therapeutic landscape of MM.

#### 3.4.3. Prostate-Specific Membrane Antigen (PSMA)

Beyond its well-established role in prostate cancer, PSMA expression has also been identified in various solid tumors, including breast cancer, renal cell carcinoma, glioblastoma, and hepatocellular carcinoma. More recently, studies have explored the potential role of PSMA PET imaging in hematological malignancies, such as lymphoma and MM, primarily based on the expression of the FOLH1 gene, which encodes PSMA [132]. In particular, AML and DLBCL have been reported to express FOLH1, suggesting that PSMA PET imaging could offer diagnostic value in these settings. Case reports have further illustrated the potential of PSMA-targeted imaging in hematologic cancers. For instance, Kanthan et al. (2016) described a case of follicular lymphoma identified using PSMA PET, while Miceli et al. (2021) reported PSMA avidity in a patient with HL [133,134]. Beyond lymphomas, PSMA PET imaging has also shown potential in MM. A recent case study reported PSMA avidity in bone lesions of a patient with MM, indicating that PSMA PET could help in disease evaluation [135]. Despite these findings, the clinical application of PSMA PET in hematological malignancies remains exploratory. Large-scale studies are required to determine its true diagnostic and therapeutic value, as well as its specificity in distinguishing malignant from benign processes in the hematopoietic system. Until then, PSMA PET should be considered an emerging but unvalidated tool in hematology, warranting further research to define its utility in comparison to established imaging modalities.

#### 3.4.4. The Place of Combination Therapies in Hematological Malignancies

The integration of targeted radiotherapy (TRT) with other modalities has shown promising synergies in hematological malignancies. As noted in this manuscript, CS1-directed radioligand therapy increases CD8⁺ T-cell infiltration and PD-L1 expression, suggesting the potential for combining TRT with immunotherapies to enhance anti-tumor responses [126]. TRT, combined with immune checkpoint inhibitors, could boost tumor immunogenicity and prepare the tumor microenvironment for immune-mediated destruction. Additionally, combining TRT with CAR-T cells or bispecific antibodies is under investigation, particularly for relapsed/refractory diseases, where antigen escape remains a major challenge [136]. However, such a combination remains to be evaluated in hematological malignancies.

An emerging approach involves combining TRT with DNA damage response inhibitors, such as PARP inhibitors, to enhance the cytotoxic effects of alpha-emitting radionuclides like ^225^Ac. However, in vivo validation is needed [60]. Among the limited combination therapies studied, CS1 and CD37 are two targets that have been explored and deserve further attention, given their potential to enhance treatment efficacy. As theranostics advances, rational combination strategies will be crucial for optimizing outcomes in hematologic cancers.

## 4. Challenges and Future Directions

Despite significant advancements in theranostic approaches for hematological malignancies, several challenges continue to limit their widespread clinical adoption. Key obstacles include antigen heterogeneity, logistical and regulatory hurdles, radionuclide production bottlenecks, supply chain challenges, and cost-effectiveness concerns. Overcoming these barriers is essential to fully unlock the potential of TRT and expand its role in precision hematology.

A major limitation in TRT lies in the dynamic expression of target antigens. Hematological malignancies often undergo antigenic drift, downregulation, or complete loss of antigens due to selective pressures from prior treatments. This is notably observed in post-CAR-T relapses, where CD19 expression diminishes [61]. Dual-targeting strategies, such as CD20/CD37 or CD33/CD123 combinations, alongside the integration of PET-based imaging for real-time antigen monitoring, could improve patient selection and enhance treatment efficacy.

Expanding the range of targets is also pivotal. Emerging markers like CD70, PSMA, and SST2R offer promising opportunities to broaden theranostic applications in hematologic oncology. While β-emitters (e.g., ^177^Lu, ^90^Y) have demonstrated efficacy, their limited tissue penetration can hinder the treatment of micrometastatic disease or bone marrow-residing tumor cells. In contrast, α-emitters (^225^Ac, ^212^Pb, ^211^At) provide LET, inducing potent cytotoxic effects with reduced off-target damage. However, their clinical translation is challenged by issues in production, biodistribution, and dosimetry standardization. Innovations in chelator chemistry, radiolabeled antibody stabilization, and combination therapies—such as pairing α therapy with DNA damage response inhibitors (e.g., PARP inhibitors)—may optimize therapeutic efficacy while minimizing systemic toxicity.

Beyond scientific and clinical challenges, the integration of theranostics into routine practice faces economic, regulatory, and logistical barriers. While radioligand therapy is gaining traction in hematologic malignancies, its commercialization remains limited due to high costs, specialized infrastructure required for radionuclide production and distribution, and disparities in access across different regions. Currently, Europe and North America dominate this market, but increasing investments in the Asia-Pacific region are driving global expansion.

Radionuclide production remains a critical bottleneck, as the availability of key isotopes such as ^177^Lu and ^225^Ac is restricted to specialized facilities, often concentrated in specific regions. This leads to supply chain limitations and uneven access across countries. Additionally, the high cost of radionuclide production, coupled with logistical challenges in maintaining the integrity of radiopharmaceuticals during transit, exacerbates cost issues. These hurdles, along with complex regulatory frameworks and a lack of standardized dosimetry, hinder the widespread clinical implementation of TRT. Addressing these issues requires scalable, cost-effective manufacturing solutions, as well as streamlined production and distribution strategies to ensure equitable access to theranostic therapies.

Optimizing patient stratification remains a key priority for refining theranostic approaches in clinical practice. Personalized treatment regimens, based on biomarkers, genetic profiling, and molecular imaging, will be critical for identifying patients most likely to benefit from specific therapies. In this context, artificial intelligence (AI) is emerging as a valuable tool for optimizing dosimetry, predicting treatment responses, and refining molecular target selection. AI-driven models could enhance patient selection and therapy personalization, potentially increasing the effectiveness of TRT. However, despite its promise, AI applications in theranostics remain in early-stage development, requiring rigorous clinical validation, regulatory approval, and integration into standard practice. Further research is needed to assess whether adaptive AI can reliably enhance clinical decision-making and whether generative AI can accelerate the discovery of novel radiopharmaceuticals.

In addition to technological advancements, exploring combination strategies with immunotherapies offers new avenues for improving treatment outcomes. Combining TRT with CAR-T cells, immune checkpoint inhibitors, and bispecific antibodies holds significant promise. Emerging data suggest that TRT can modulate the tumor microenvironment, potentially enhancing immune-mediated responses and reducing antigen escape. Additionally, advancements in molecular imaging using ^89^Zr, ^68^Ga, and ^64^Cu-labeled antibodies will refine patient selection and support adaptive treatment planning, ensuring maximal therapeutic benefit with minimal toxicity.

As the field evolves, a multidisciplinary approach will be key to overcoming current limitations and fully harnessing the transformative potential of theranostics in hematologic malignancies.

## 5. Conclusions

Advances in nuclear medicine are transforming the therapeutic landscape of hematological malignancies, with key targets in B-cell lymphomas, leukemias, and multiple myeloma now explored for theranostic applications. Both β-emitters (e.g., ^177^Lu) and α-emitters (e.g., ^225^Ac, ^212^Pb) have shown the potential to overcome therapeutic resistance and enhance tumor selectivity, offering new avenues for patients with refractory or relapsed disease. Beyond established targets, the investigation of emerging biomarkers—such as SST2R and PSMA—highlights opportunities for drug repositioning and broadens the therapeutic arsenal in hematologic cancers.

To fully harness the potential of theranostics in clinical practice, future efforts should focus on optimizing combination strategies, particularly by integrating TRT with CAR-T therapy, bispecific antibodies, and immune checkpoint inhibitors to enhance treatment efficacy and mitigate antigen escape. Additionally, refining dosimetry protocols and standardizing personalized dose calculations will be essential to improve tumor control while minimizing toxicity. Given the need for robust clinical validation, upcoming trials should prioritize randomized controlled trials to establish efficacy and safety, while also incorporating real-world evidence studies to assess long-term outcomes, accessibility, and cost-effectiveness in broader patient populations. Adaptive clinical trial designs, which allow treatment adjustments based on imaging and biomarker-driven responses, could further accelerate the integration of these therapies into standard practice. Expanding international collaborations through multicenter clinical trials and regulatory harmonization will also be key to facilitating the widespread adoption of theranostic approaches.

As first-in-human and large-scale clinical trials progress, their outcomes will be pivotal in defining the clinical utility of these agents and determining their integration into standard-of-care protocols. The synergy between nuclear medicine, immunotherapy, and AI-driven patient selection holds significant promise in shaping the future of precision hematology, paving the way for more personalized and effective treatment strategies in hematological malignancies.

## Figures and Tables

**Table 1:** Radiopharmaceuticals currently in development for tumor imaging and RIT in hematological cancers.

Cancer Type	Cellular Target	Targeting Compound	Radioisotope	Tumor Model	Clinical Trial	References
B-cell Lymphomas	CD20	ibritumomab tiuxetan (Zevalin)	^90^Y	/	NHL patients (FDA approved in 2002)	[12,13,15,16]
		tositumomab	^131^I	/	NHL patients (FDA approved in 2003)	[13,14]
		rituximab	^177^Lu, ^89^Zr, ^64^Cu	/	Follicular, Mantle and Marginal Lymphoma (Phase I/II); NHL (Phase I)	[41,42,43]
		ofatumumab	^177^Lu, ^225^Ac, ^89^Zr	Raji-cell or 38C13-hCD20	/	[44,45,46,47,48]
	CD37	Lilotomab satetraxetan (Betalutin^®^)	^177^Lu	DOHH2 or Raji-cell	Indolent NHL (First in human [FIH])	[52,53,54,55,56,57]
		NNV003	^177^Lu, ^89^Zr, ^212^Pb	REC-1, REC1 B, RAMOS, Daudi or MEC-2	/	[58,59,127]
	CD22	huRFB4	^177^Lu	Raji-cell	/	[64,65]
		epratuzumab, G5/44	^211^At	Ramos	/	[66]
	CD74	LL1	^213^Bi	Raji-cell	/	[72]
Myeloid and Lymphoid Leukemias	CD33	lintuzumab	^89^Zr, ^227^Th, ^225^Ac	OCI-AML3, HL-60 or U937	AML patients (FIH)	[76,78,79,80,81]
		CD33-targeting Nbs	^99m^Tc	THP-1	/	[77]
	CD123	CD123-targeting mAbs	^211^At	MOLM-13	/	[87]
	CD45	apamistamab	^131^I	/	AML patients (Phase III)	[89]
		BC8	^90^Y, ^131^I	/	MM patients (Phase I); B-NHL, T-NHL, and HL patients (Phase I)	[90,91]
	CXCR4	pentixather	^177^Lu, ^90^Y	Daudi	MM patients (FIH); AML patients (Phase I/II)	[95,96,97,98]
Multiple Myeloma	CD38	isatuximab	^89^Zr	MM.1S or K562	/	[104]
		daratumumab	^89^Zr, ^212^Pb, ^225^Ac, ^177^Lu	RPMI 8226, MOLP-8, OPM-2, NCI-H929 or MM1-S	MM patients (Phase II)	[105,113,116]
		CD3813 Nb	^99m^Tc, ^68^Ga	Ramos	/	[106,108]
		Nb1053	^18^F, ^68^Ga	MM.1S	/	[107,109]
		AJ206	^68^Ga	MOLP8, MM.1S or patient cells	/	[110]
		SL022-GGS	^64^Cu	MM.1S	/	[111]
		CD38-targeting sdAbs	^177^Lu	RPMI 8226	/	[112]
		MOR03087	^213^Bi	OPM2	/	[114]
		OKT10-B10	^211^At	OPM-2 or NCI-H929	/	[115]
	BCMA	BCMAh230430	^89^Zr, ^177^Lu	MM.1S or KYSE520	/	[121]
		BCMA-B10	^211^At	MM1R or NCI-H929	/	[122]
	CS1	elotuzumab	^89^Zr	MM.1S	/	[125]
		CS1-targeting sdAbs	^225^Ac	5T33MM or 5TGM1	/	[126]

## Abbreviations

The following abbreviations are used in this manuscript:

ADCC	Antibody-dependent cellular cytotoxicity
ADCs	Antibody-drug conjugates
AML	Acute myeloid leukemia
ALL	Acute lymphoblastic leukemia
B-ALL	B-cell acute lymphoblastic leukemia
BCR	B-cell receptor
BCMA	B-cell maturation antigen
BiTEs	Bispecific T-cell engagers
BPDCN	Blastic plasmacytoid dendritic cell neoplasm
CAR	Chimeric antigen receptor
CLL	Chronic lymphocytic leukemia
CML	Chronic myeloid leukemia
CRS	Cytokine release syndrome
CD	Cluster of Differentiation
dCR	Deep complete remission
HSCT	Hematopoietic stem cell transplantation
LET	Linear energy transfer
LSCs	Leukemic stem cells
MM	Multiple myeloma
mAbs	Monoclonal antibodies
MRD	Minimal residual disease
NHL	Non-Hodgkin lymphoma
NK	Natural killer
PSMA	Prostate-Specific Membrane Antigen
R/R	Relapsed/refractory
SLAMF7	Signaling Lymphocytic Activation Molecule Family Member 7
SSTR	Somatostatin receptors
TNF	Tumor necrosis factor
TRT	Targeted radiotherapy

## References

[1] Taylor J., Xiao W., Abdel-Wahab O. (2017). Diagnosis and Classification of Hematologic Malignancies on the Basis of Genetics. Blood.

[2] Zhang N., Wu J., Wang Q., Liang Y., Li X., Chen G., Ma L., Liu X., Zhou F. (2023). Global Burden of Hematologic Malignancies and Evolution Patterns over the Past 30 Years. Blood Cancer J..

[3] Brown G. (2022). Lessons to Cancer from Studies of Leukemia and Hematopoiesis. Front. Cell Dev. Biol..

[4] Awais M., Abdal M.N., Akram T., Alasiry A., Marzougui M., Masood A. (2024). An Efficient Decision Support System for Leukemia Identification Utilizing Nature-Inspired Deep Feature Optimization. Front. Oncol..

[5] Alaggio R., Amador C., Anagnostopoulos I., Attygalle A.D., Araujo I.B.d.O., Berti E., Bhagat G., Borges A.M., Boyer D., Calaminici M. (2022). The 5th Edition of the World Health Organization Classification of Haematolymphoid Tumours: Lymphoid Neoplasms. Leukemia.

[6] Rajkumar S.V. (2024). Multiple Myeloma: 2024 Update on Diagnosis, Risk-Stratification, and Management. Am. J. Hematol..

[7] Sochacka-Ćwikła A., Mączyński M., Regiec A. (2021). FDA-Approved Drugs for Hematological Malignancies—The Last Decade Review. Cancers.

[8] Weber W.A., Barthel H., Bengel F., Eiber M., Herrmann K., Schäfers M. (2023). What Is Theranostics?. J. Nucl. Med..

[9] Di Franco M., Zanoni L., Fortunati E., Fanti S., Ambrosini V. (2024). Radionuclide Theranostics in Neuroendocrine Neoplasms: An Update. Curr. Oncol. Rep..

[10] Yordanova A., Eppard E., Kürpig S., Bundschuh R.A., Schönberger S., Gonzalez-Carmona M., Feldmann G., Ahmadzadehfar H., Essler M. (2017). Theranostics in Nuclear Medicine Practice. Onco Targets Ther..

[11] Echavidre W., Fagret D., Faraggi M., Picco V., Montemagno C. (2023). Recent Pre-Clinical Advancements in Nuclear Medicine: Pioneering the Path to a Limitless Future. Cancers.

[12] Grillo-López A.J. (2002). Zevalin: The First Radioimmunotherapy Approved for the Treatment of Lymphoma. Expert Rev. Anticancer Ther..

[13] Jacene H.A., Filice R., Kasecamp W., Wahl R.L. (2007). Comparison of 90Y-Ibritumomab Tiuxetan and 131I-Tositumomab in Clinical Practice. J. Nucl. Med..

[14] Cheung M.C., MacEachern J.A., Haynes A.E., Meyer R.M., Imrie K. (2009). 131I–Tositumomab in Lymphoma. Curr. Oncol..

[15] Witzig T.E., Flinn I.W., Gordon L.I., Emmanouilides C., Czuczman M.S., Saleh M.N., Cripe L., Wiseman G., Olejnik T., Multani P.S. (2002). Treatment with Ibritumomab Tiuxetan Radioimmunotherapy in Patients with Rituximab-Refractory Follicular Non-Hodgkin’s Lymphoma. J. Clin. Oncol..

[16] Witzig T.E., Gordon L.I., Cabanillas F., Czuczman M.S., Emmanouilides C., Joyce R., Pohlman B.L., Bartlett N.L., Wiseman G.A., Padre N. (2002). Randomized Controlled Trial of Yttrium-90-Labeled Ibritumomab Tiuxetan Radioimmunotherapy versus Rituximab Immunotherapy for Patients with Relapsed or Refractory Low-Grade, Follicular, or Transformed B-Cell Non-Hodgkin’s Lymphoma. J. Clin. Oncol..

[17] Omer M.H., Shafqat A., Ahmad O., Alkattan K., Yaqinuddin A., Damlaj M. (2023). Bispecific Antibodies in Hematological Malignancies: A Scoping Review. Cancers.

[18] Mansoori S., Noei A., Maali A., Seyed-Motahari S.S., Sharifzadeh Z. (2024). Recent Updates on Allogeneic CAR-T Cells in Hematological Malignancies. Cancer Cell Int..

[19] Dun Y., Huang G., Liu J., Wei W. (2022). ImmunoPET Imaging of Hematological Malignancies: From Preclinical Promise to Clinical Reality. Drug Discov. Today.

[20] Zamanian M., Albano D., Treglia G., Rizzo A., Abedi I. (2024). The Clinical Role of CXCR4-Targeted PET on Lymphoproliferative Disorders: A Systematic Review. J. Clin. Med..

[21] Alqahtani F.F. (2023). SPECT/CT and PET/CT, Related Radiopharmaceuticals, and Areas of Application and Comparison. Saudi Pharm. J..

[22] Das T., Banerjee S. (2016). Theranostic Applications of Lutetium-177 in Radionuclide Therapy. Curr. Radiopharm..

[23] Kang L., Li C., Rosenkrans Z.T., Huo N., Chen Z., Ehlerding E.B., Huo Y., Ferreira C.A., Barnhart T.E., Engle J.W. (2021). CD38-Targeted Theranostics of Lymphoma with 89Zr/177Lu-Labeled Daratumumab. Adv. Sci..

[24] Mondello P., Cuzzocrea S., Navarra M., Mian M. (2015). 90 Y-Ibritumomab Tiuxetan: A Nearly Forgotten Opportunity. Oncotarget.

[25] Haberkorn U., Giesel F., Morgenstern A., Kratochwil C. (2017). The Future of Radioligand Therapy: α, β, or Both?. J. Nucl. Med..

[26] Bidkar A.P., Zerefa L., Yadav S., VanBrocklin H.F., Flavell R.R. (2024). Actinium-225 Targeted Alpha Particle Therapy for Prostate Cancer. Theranostics.

[27] Ruigrok E.A.M., Tamborino G., de Blois E., Roobol S.J., Verkaik N., De Saint-Hubert M., Konijnenberg M.W., van Weerden W.M., de Jong M., Nonnekens J. (2022). In Vitro Dose Effect Relationships of Actinium-225- and Lutetium-177-Labeled PSMA-I&T. Eur. J. Nucl. Med. Mol. Imaging.

[28] Eychenne R., Chérel M., Haddad F., Guérard F., Gestin J.-F. (2021). Overview of the Most Promising Radionuclides for Targeted Alpha Therapy: The “Hopeful Eight”. Pharmaceutics.

[29] Dawicki W., Allen K.J.H., Jiao R., Malo M.E., Helal M., Berger M.S., Ludwig D.L., Dadachova E. (2019). Daratumumab-225Actinium Conjugate Demonstrates Greatly Enhanced Antitumor Activity against Experimental Multiple Myeloma Tumors. Oncoimmunology.

[30] Juzeniene A., Stenberg V.Y., Bruland Ø.S., Revheim M.-E., Larsen R.H. (2023). Dual Targeting with 224Ra/212Pb-Conjugates for Targeted Alpha Therapy of Disseminated Cancers: A Conceptual Approach. Front. Med..

[31] Dahle J., Saidi A., Stallons T., Heyerdahl H., Repetto-Llamazares A., Torgue J. (2022). Abstract 5432: Targeted Alpha Therapy with 212Pb-NNV003 in Treatment of NHL. Cancer Res..

[32] Durand-Panteix S., Monteil J., Sage M., Garot A., Clavel M., Saidi A., Torgue J., Cogne M., Quelven I. (2021). Preclinical Study of 212Pb Alpha-Radioimmunotherapy Targeting CD20 in Non-Hodgkin Lymphoma. Br. J. Cancer.

[33] Van Laere C., Koole M., Deroose C.M., de Voorde M.V., Baete K., Cocolios T.E., Duchemin C., Ooms M., Cleeren F. (2024). Terbium Radionuclides for Theranostic Applications in Nuclear Medicine: From Atom to Bedside. Theranostics.

[34] Ku A., Facca V.J., Cai Z., Reilly R.M. (2019). Auger Electrons for Cancer Therapy—A Review. EJNMMI Radiopharm. Chem..

[35] Zukotynski K., Jadvar H., Capala J., Fahey F. (2016). Targeted Radionuclide Therapy: Practical Applications and Future Prospects. Biomark. Cancer.

[36] Aparicio-Pérez C., Carmona M., Benabdellah K., Herrera C. (2023). Failure of ALL Recognition by CAR T Cells: A Review of CD 19-Negative Relapses after Anti-CD 19 CAR-T Treatment in B-ALL. Front. Immunol..

[37] Patel R.B., Hernandez R., Carlson P., Grudzinski J., Bates A.M., Jagodinsky J.C., Erbe A., Marsh I.R., Arthur I., Aluicio-Sarduy E. (2021). Low-Dose Targeted Radionuclide Therapy Renders Immunologically Cold Tumors Responsive to Immune Checkpoint Blockade. Sci. Transl. Med..

[38] Khazaei Monfared Y., Heidari P., Klempner S.J., Mahmood U., Parikh A.R., Hong T.S., Strickland M.R., Esfahani S.A. (2023). DNA Damage by Radiopharmaceuticals and Mechanisms of Cellular Repair. Pharmaceutics.

[39] Morris E.C., Neelapu S.S., Giavridis T., Sadelain M. (2022). Cytokine Release Syndrome and Associated Neurotoxicity in Cancer Immunotherapy. Nat. Rev. Immunol..

[40] Philippa J., Cheetham M.D., Daniel P., Petrylak M.D. (2012). Alpha Particles as Radiopharmaceuticals in the Treatment of Bone Metastases: Mechanism of Action of Radium-223 Chloride (Alpharadin) and Radiation. Oncology.

[41] Forrer F., Oechslin-Oberholzer C., Campana B., Herrmann R., Maecke H.R., Mueller-Brand J., Lohri A. (2013). Radioimmunotherapy with 177Lu-DOTA-Rituximab: Final Results of a Phase I/II Study in 31 Patients with Relapsing Follicular, Mantle Cell, and Other Indolent B-Cell Lymphomas. J. Nucl. Med..

[42] Yadav M.P., Singla S., Thakral P., Ballal S., Bal C. (2016). Dosimetric Analysis of 177Lu-DOTA-Rituximab in Patients with Relapsed/Refractory Non-Hodgkin’s Lymphoma. Nucl. Med. Commun..

[43] Edamadaka Y., Parghane R.V., Sahu S., Lad S., Kamaldeep, Wanage G., Shanmukhaiah C., Kulkarni V., Basu S. (2024). Internal Dosimetry and Biodistribution of Indigenously Prepared 177 Lu-DOTA-Rituximab in Lymphoma and Other Hematological Malignancies Treated with Rituximab. Nucl. Med. Commun..

[44] Shim K., Longtine M.S., Abou D.S., Hoegger M.J., Laforest R.S., Thorek D.L.J., Wahl R.L. (2023). Cure of Disseminated Human Lymphoma with [177Lu]Lu-Ofatumumab in a Preclinical Model. J. Nucl. Med..

[45] Longtine M.S., Shim K., Hoegger M.J., Benabdallah N., Abou D.S., Thorek D.L.J., Wahl R.L. (2023). Cure of Disseminated Human Lymphoma with [225Ac]Ac-Ofatumumab in a Preclinical Model. J. Nucl. Med..

[46] Rouhollahi Z., Aghamiri S.M.R., Yousefnia H., Alirezapour B., Moghaddasi A., Zolghadri S. (2024). Preclinical Aspects of [89Zr]Zr-DFO-Rituximab: A High Potential Agent for Immuno-PET Imaging. Curr. Radiopharm..

[47] Zettlitz K.A., Salazar F.B., Yamada R.E., Trinh K.R., Vasuthasawat A., Timmerman J.M., Morrison S.L., Wu A.M. (2022). 89Zr-ImmunoPET Shows Therapeutic Efficacy of Anti-CD20-IFNα Fusion Protein in a Murine B-Cell Lymphoma Model. Mol. Cancer Ther..

[48] Abou D.S., Longtine M., Fears A., Benabdallah N., Unnerstall R., Johnston H., Shim K., Hasson A., Zhang H., Ulmert D. (2023). Evaluation of Candidate Theranostics for 227Th/89Zr Paired Radioimmunotherapy of Lymphoma. J. Nucl. Med..

[49] Lee I., Lim I., Lee K.C., Kang H.J., Lim S.M. (2023). 64 Cu-DOTA-Rituximab PET/CT of B-Cell Non-Hodgkin Lymphoma for Imaging the CD20 Expression. Clin. Nucl. Med..

[50] Bobrowicz M., Kubacz M., Slusarczyk A., Winiarska M. (2020). CD37 in B Cell Derived Tumors—More than Just a Docking Point for Monoclonal Antibodies. Int. J. Mol. Sci..

[51] Lapalombella R., Yeh Y.-Y., Wang L., Ramanunni A., Rafiq S., Jha S., Staubli J., Lucas D.M., Mani R., Herman S.E.M. (2012). Tetraspanin CD37 Directly Mediates Transduction of Survival and Apoptotic Signals. Cancer Cell.

[52] Malenge M.M., Patzke S., Ree A.H., Stokke T., Ceuppens P., Middleton B., Dahle J., Repetto-Llamazares A.H.V. (2020). 177Lu-Lilotomab Satetraxetan Has the Potential to Counteract Resistance to Rituximab in Non-Hodgkin Lymphoma. J. Nucl. Med..

[53] Blakkisrud J., Løndalen A., Martinsen A.C.T., Dahle J., Holtedahl J.E., Bach-Gansmo T., Holte H., Kolstad A., Stokke C. (2017). Tumor-Absorbed Dose for Non-Hodgkin Lymphoma Patients Treated with the Anti-CD37 Antibody Radionuclide Conjugate 177Lu-Lilotomab Satetraxetan. J. Nucl. Med..

[54] Blakkisrud J., Holtedahl J.E., Løndalen A., Dahle J., Bach-Gansmo T., Holte H., Nygaard S., Kolstad A., Stokke C. (2018). Biodistribution and Dosimetry Results from a Phase 1 Trial of Therapy with the Antibody-Radionuclide Conjugate 177Lu-Lilotomab Satetraxetan. J. Nucl. Med..

[55] Kolstad A., Illidge T., Bolstad N., Spetalen S., Madsbu U., Stokke C., Blakkisrud J., Løndalen A., O’Rourke N., Beasley M. (2020). Phase 1/2a Study of 177Lu-Lilotomab Satetraxetan in Relapsed/Refractory Indolent Non-Hodgkin Lymphoma. Blood Adv..

[56] Pichard A., Marcatili S., Karam J., Constanzo J., Ladjohounlou R., Courteau A., Jarlier M., Bonnefoy N., Patzke S., Stenberg V. (2020). The Therapeutic Effectiveness of 177Lu-Lilotomab in B-Cell Non-Hodgkin Lymphoma Involves Modulation of G2/M Cell Cycle Arrest. Leukemia.

[57] Løndalen A., Blakkisrud J., Revheim M.-E., Dahle J., Kolstad A., Stokke C. (2022). FDG PET/CT and Dosimetric Studies of 177Lu-Lilotomab Satetraxetan in a First-in-Human Trial for Relapsed Indolent Non-Hodgkin Lymphoma-Are We Hitting the Target?. Mol. Imaging Biol..

[58] Maaland A.F., Saidi A., Torgue J., Heyerdahl H., Stallons T.A.R., Kolstad A., Dahle J. (2020). Targeted Alpha Therapy for Chronic Lymphocytic Leukaemia and Non-Hodgkin’s Lymphoma with the Anti-CD37 Radioimmunoconjugate 212Pb-NNV003. PLoS ONE.

[59] Maaland A.F., Heyerdahl H., O’Shea A., Eiriksdottir B., Pascal V., Andersen J.T., Kolstad A., Dahle J. (2019). Targeting B-Cell Malignancies with the Beta-Emitting Anti-CD37 Radioimmunoconjugate 177Lu-NNV003. Eur. J. Nucl. Med. Mol. Imaging.

[60] Giesen D., Hooge M.N.L., Nijland M., Heyerdahl H., Dahle J., de Vries E.G.E., Pool M. (2022). 89Zr-PET Imaging to Predict Tumor Uptake of 177Lu-NNV003 Anti-CD37 Radioimmunotherapy in Mouse Models of B Cell Lymphoma. Sci. Rep..

[61] Malenge M.M., Maaland A.F., Repetto-Llamazares A., Middleton B., Nijland M., Visser L., Patzke S., Heyerdahl H., Kolstad A., Stokke T. (2022). Anti-CD37 Radioimmunotherapy with 177Lu-NNV003 Synergizes with the PARP Inhibitor Olaparib in Treatment of Non-Hodgkin’s Lymphoma in Vitro. PLoS ONE.

[62] Xu J., Luo W., Li C., Mei H. (2023). Targeting CD22 for B-Cell Hematologic Malignancies. Exp. Hematol. Oncol..

[63] Schultz L., Gardner R. (2019). Mechanisms of and Approaches to Overcoming Resistance to Immunotherapy. Hematol. Am. Soc. Hematol. Educ. Program..

[64] Yurkiewicz I.R., Muffly L., Liedtke M. (2018). Inotuzumab Ozogamicin: A CD22 mAb–Drug Conjugate for Adult Relapsed or Refractory B-Cell Precursor Acute Lymphoblastic Leukemia. Drug Des. Devel Ther..

[65] Weber T., Bötticher B., Arndt M.A.E., Mier W., Sauter M., Exner E., Keller A., Krämer S., Leotta K., Wischnjow A. (2016). Preclinical Evaluation of a Diabody-Based (177)Lu-Radioimmunoconjugate for CD22-Directed Radioimmunotherapy in a Non-Hodgkin Lymphoma Mouse Model. Cancer Lett..

[66] Weber T., Bötticher B., Mier W., Sauter M., Krämer S., Leotta K., Keller A., Schlegelmilch A., Grosse-Hovest L., Jäger D. (2016). High Treatment Efficacy by Dual Targeting of Burkitt’s Lymphoma Xenografted Mice with a (177)Lu-Based CD22-Specific Radioimmunoconjugate and Rituximab. Eur. J. Nucl. Med. Mol. Imaging.

[67] Laszlo G.S., Sandmaier B.M., Kehret A.R., Orozco J.J., Hamlin D.K., Dexter S.L., Lim S.Y.T., Cole F.M., Huo J., Wilbur D.S. (2023). [211At]Astatine-Based Anti-CD22 Radioimmunotherapy For B-Cell Malignancies. Leuk. Lymphoma.

[68] Niu J., Qiu H., Xiang F., Zhu L., Yang J., Huang C., Zhou K., Tong Y., Cai Y., Dong B. (2023). CD19/CD22 Bispecific CAR-T Cells for MRD-Positive Adult B Cell Acute Lymphoblastic Leukemia: A Phase I Clinical Study. Blood Cancer J..

[69] Stein R., Mattes M.J., Cardillo T.M., Hansen H.J., Chang C.-H., Burton J., Govindan S., Goldenberg D.M. (2007). CD74: A New Candidate Target for the Immunotherapy of B-Cell Neoplasms. Clin. Cancer Res..

[70] Hertlein E., Triantafillou G., Sass E.J., Hessler J.D., Zhang X., Jarjoura D., Lucas D.M., Muthusamy N., Goldenberg D.M., Lee R.J. (2010). Milatuzumab Immunoliposomes Induce Cell Death in CLL by Promoting Accumulation of CD74 on the Surface of B Cells. Blood.

[71] Le Q., Tang T., Leonti A., Castro S., McKay C.N., Perkins L., Pardo L., Kirkey D., Hylkema T., Call L. (2023). Preclinical Studies Targeting CD74 with STRO-001 Antibody-Drug Conjugate in Acute Leukemia. Blood Adv..

[72] Li X., Abrahams C., Yu A., Embry M., Henningsen R., DeAlmeida V., Matheny S., Kline T., Yam A., Stafford R. (2023). Targeting CD74 in B-Cell Non-Hodgkin Lymphoma with the Antibody-Drug Conjugate STRO-001. Oncotarget.

[73] Michel R.B., Rosario A.V., Brechbiel M.W., Jackson T.J., Goldenberg D.M., Mattes M.J. (2003). Experimental Therapy of Disseminated B-Cell Lymphoma Xenografts with 213Bi-Labeled Anti-CD74. Nucl. Med. Biol..

[74] Linenberger M.L. (2005). CD33-Directed Therapy with Gemtuzumab Ozogamicin in Acute Myeloid Leukemia: Progress in Understanding Cytotoxicity and Potential Mechanisms of Drug Resistance. Leukemia.

[75] Liu Y., Wang S., Schubert M.-L., Lauk A., Yao H., Blank M.F., Cui C., Janssen M., Schmidt C., Göllner S. (2022). CD33-Directed Immunotherapy with Third-Generation Chimeric Antigen Receptor T Cells and Gemtuzumab Ozogamicin in Intact and CD33-Edited Acute Myeloid Leukemia and Hematopoietic Stem and Progenitor Cells. Int. J. Cancer.

[76] Maakaron J.E., Rogosheske J., Long M., Bachanova V., Mims A.S. (2021). CD33-Targeted Therapies: Beating the Disease or Beaten to Death?. J. Clin. Pharmacol..

[77] Allen K.J.H., Jiao R., Li J., Beckford-Vera D.R., Dadachova E. (2022). In Vitro and In Vivo Characterization of 89Zirconium-Labeled Lintuzumab Molecule. Molecules.

[78] Romão E., Krasniqi A., Maes L., Vandenbrande C., Sterckx Y.G.-J., Stijlemans B., Vincke C., Devoogdt N., Muyldermans S. (2020). Identification of Nanobodies against the Acute Myeloid Leukemia Marker CD33. Int. J. Mol. Sci..

[79] Hagemann U.B., Wickstroem K., Wang E., Shea A.O., Sponheim K., Karlsson J., Bjerke R.M., Ryan O.B., Cuthbertson A.S. (2016). In Vitro and In Vivo Efficacy of a Novel CD33-Targeted Thorium-227 Conjugate for the Treatment of Acute Myeloid Leukemia. Mol. Cancer Ther..

[80] Hagemann U.B., Wickstroem K., Hammer S., Bjerke R.M., Zitzmann-Kolbe S., Ryan O.B., Karlsson J., Scholz A., Hennekes H., Mumberg D. (2020). Advances in Precision Oncology: Targeted Thorium-227 Conjugates As a New Modality in Targeted Alpha Therapy. Cancer Biother. Radiopharm..

[81] Garg R., Allen K.J.H., Dawicki W., Geoghegan E.M., Ludwig D.L., Dadachova E. (2021). 225Ac-Labeled CD33-Targeting Antibody Reverses Resistance to Bcl-2 Inhibitor Venetoclax in Acute Myeloid Leukemia Models. Cancer Med..

[82] Rosenblat T.L., McDevitt M.R., Carrasquillo J.A., Pandit-Taskar N., Frattini M.G., Maslak P.G., Park J.H., Douer D., Cicic D., Larson S.M. (2022). Treatment of Patients with Acute Myeloid Leukemia with the Targeted Alpha-Particle Nanogenerator Actinium-225-Lintuzumab. Clin. Cancer Res..

[83] Testa U., Pelosi E., Castelli G. (2019). CD123 as a Therapeutic Target in the Treatment of Hematological Malignancies. Cancers.

[84] El Achi H., Dupont E., Paul S., Khoury J.D. (2020). CD123 as a Biomarker in Hematolymphoid Malignancies: Principles of Detection and Targeted Therapies. Cancers.

[85] Pemmaraju N., Konopleva M. (2020). Approval of Tagraxofusp-Erzs for Blastic Plasmacytoid Dendritic Cell Neoplasm. Blood Adv..

[86] Zanotta S., Galati D., De Filippi R., Pinto A. (2024). Breakthrough in Blastic Plasmacytoid Dendritic Cell Neoplasm Cancer Therapy Owing to Precision Targeting of CD123. Int. J. Mol. Sci..

[87] Daver N.G., Montesinos P., DeAngelo D.J., Wang E.S., Papadantonakis N., Todisco E., Sweet K.L., Pemmaraju N., Lane A.A., Torres-Miñana L. (2024). Pivekimab Sunirine (IMGN632), a Novel CD123-Targeting Antibody-Drug Conjugate, in Relapsed or Refractory Acute Myeloid Leukaemia: A Phase 1/2 Study. Lancet Oncol..

[88] Laszlo G.S., Orozco J.J., Kehret A.R., Lunn M.C., Huo J., Hamlin D.K., Scott Wilbur D., Dexter S.L., Comstock M.L., O’Steen S. (2022). Development of [211At]Astatine-Based Anti-CD123 Radioimmunotherapy for Acute Leukemias and Other CD123+ Malignancies. Leukemia.

[89] Ye N., Cai J., Dong Y., Chen H., Bo Z., Zhao X., Xia M., Han M. (2022). A Multi-Omic Approach Reveals Utility of CD45 Expression in Prognosis and Novel Target Discovery. Front. Genet..

[90] Gyurkocza B., Nath R., Seropian S., Choe H., Litzow M.R., Abboud C., Koshy N., Stiff P., Tomlinson B., Abhyankar S. (2025). Randomized Phase III SIERRA Trial of 131I-Apamistamab Before Allogeneic Hematopoietic Cell Transplantation Versus Conventional Care for Relapsed/Refractory AML. J. Clin. Oncol..

[91] Tuazon S.A., Sandmaier B.M., Gooley T.A., Fisher D.R., Holmberg L.A., Becker P.S., Lundberg S.J., Orozco J.J., Gopal A.K., Till B.G. (2021). 90Y-Labeled Anti-CD45 Antibody Allogeneic Hematopoietic Cell Transplantation for High-Risk Multiple Myeloma. Bone Marrow Transpl..

[92] Cassaday R.D., Press O.W., Pagel J.M., Rajendran J.G., Gooley T.A., Fisher D.R., Holmberg L.A., Miyaoka R.S., Sandmaier B.M., Green D.J. (2019). Phase I Study of a CD45-Targeted Antibody-Radionuclide Conjugate for High-Risk Lymphoma. Clin. Cancer Res..

[93] Baljinder S., Ankit W., Shekhawat A.S., Ashwin S., Malhotra P., Waheed A., Harneet K., Nisha R., Madan R., Arora S., Prasad V. (2024). CXCR4 Theranostics: A Potential Game Changer in Solid Tumors and Hematological Malignancies. Beyond Becquerel and Biology to Precision Radiomolecular Oncology: Festschrift in Honor of Richard P. Baum.

[94] Peled A., Klein S., Beider K., Burger J.A., Abraham M. (2018). Role of CXCL12 and CXCR4 in the Pathogenesis of Hematological Malignancies. Cytokine.

[95] Burger J.A., Kipps T.J. (2006). CXCR4: A Key Receptor in the Crosstalk between Tumor Cells and Their Microenvironment. Blood.

[96] Buck A.K., Serfling S.E., Lindner T., Hänscheid H., Schirbel A., Hahner S., Fassnacht M., Einsele H., Werner R.A. (2022). CXCR4-Targeted Theranostics in Oncology. Eur. J. Nucl. Med. Mol. Imaging.

[97] Herrmann K., Schottelius M., Lapa C., Osl T., Poschenrieder A., Hänscheid H., Lückerath K., Schreder M., Bluemel C., Knott M. (2016). First-in-Human Experience of CXCR4-Directed Endoradiotherapy with 177Lu- and 90Y-Labeled Pentixather in Advanced-Stage Multiple Myeloma with Extensive Intra- and Extramedullary Disease. J. Nucl. Med..

[98] Schottelius M., Osl T., Poschenrieder A., Hoffmann F., Beykan S., Hänscheid H., Schirbel A., Buck A.K., Kropf S., Schwaiger M. (2017). [177Lu]Pentixather: Comprehensive Preclinical Characterization of a First CXCR4-Directed Endoradiotherapeutic Agent. Theranostics.

[99] Braitsch K., Lorenzini T., Hefter M., Koch K., Nickel K., Peeken J.C., Götze K.S., Weber W., Allmann A., D’Alessandria C. (2025). CXCR4-Directed Endoradiotherapy with [177Lu]Pentixather Added to Total Body Irradiation for Myeloablative Conditioning in Patients with Relapsed/Refractory Acute Myeloid Leukemia. Theranostics.

[100] Christensen K.A., Fath M.A., Ewald J.T., Robles-Planells C., Graves S.A., Johnson S.S., Zacharias Z.R., Houtman J.C.D., O’Dorisio M.S., Schultz M.K. (2024). Targeting CXCR4 with [212Pb/203Pb]-Pentixather Significantly Increases Overall Survival in Small Cell Lung Cancer. bioRxiv.

[101] Kawano Y., Kushima S., Hata H., Matsuoka M. (2021). The Role of CD38 in Multiple Myeloma Cell Biology. Blood.

[102] Shen F., Shen W. (2022). Isatuximab in the Treatment of Multiple Myeloma: A Review and Comparison with Daratumumab. Technol. Cancer Res. Treat..

[103] Kikuchi T., Tsukada N., Nomura M., Kasuya Y., Oda Y., Sato K., Takei T., Ogura M., Abe Y., Suzuki K. (2023). Real-World Clinical Outcomes in Patients with Multiple Myeloma Treated with Isatuximab after Daratumumab Treatment. Ann. Hematol..

[104] Sonneveld P., Dimopoulos M.A., Boccadoro M., Quach H., Ho P.J., Beksac M., Hulin C., Antonioli E., Leleu X., Mangiacavalli S. (2024). Daratumumab, Bortezomib, Lenalidomide, and Dexamethasone for Multiple Myeloma. N. Engl. J. Med..

[105] Herrero Alvarez N., Michel A.L., Viray T.D., Mayerhoefer M.E., Lewis J.S. (2023). 89Zr-DFO-Isatuximab for CD38-Targeted ImmunoPET Imaging of Multiple Myeloma and Lymphomas. ACS Omega.

[106] Ulaner G.A., Lewis J., Landgren O. (2025). CD38-Targeted 89Zr-DFO-Daratumumab PET of Myeloma: Immuno-PET Impacting Clinical Care. J. Nucl. Med..

[107] Shi L., Chen B., Liu T., Li L., Hu B., Li C., Jia B., Wang F. (2022). 99mTc-CD3813: A Nanobody-Based Single Photon Emission Computed Tomography Radiotracer with Clinical Potential for Myeloma Imaging and Evaluation of CD38 Expression. Mol. Pharm..

[108] Wei W., Zhang D., Wang C., Zhang Y., An S., Chen Y., Huang G., Liu J. (2022). Annotating CD38 Expression in Multiple Myeloma with [18F]F-Nb1053. Mol. Pharm..

[109] Huang W., Wang T., Qiu Y., Li C., Chen B., Song L., Yang Q., Sun X., Jia B., Kang L. (2024). CD38-Specific immunoPET Imaging for Multiple Myeloma Diagnosis and Therapeutic Monitoring: Preclinical and First-in-Human Studies. Eur. J. Nucl. Med. Mol. Imaging.

[110] Wang C., Chen Y., Hou Y.N., Liu Q., Zhang D., Zhao H., Zhang Y., An S., Li L., Hou J. (2021). ImmunoPET Imaging of Multiple Myeloma with [68Ga]Ga-NOTA-Nb1053. Eur. J. Nucl. Med. Mol. Imaging.

[111] Sharma A.K., Gupta K., Mishra A., Lofland G., Marsh I., Kumar D., Ghiaur G., Imus P., Rowe S.P., Hobbs R.F. (2024). CD38-Specific Gallium-68 Labeled Peptide Radiotracer Enables Pharmacodynamic Monitoring in Multiple Myeloma with PET. Adv. Sci..

[112] Zheleznyak A., Tang R., Duncan K., Manion B., Liang K., Xu B., Vanover A., Ghai A., Prior J., Lees S. (2024). Development of New CD38 Targeted Peptides for Cancer Imaging. Mol. Imaging Biol..

[113] Duray E., Lejeune M., Baron F., Beguin Y., Devoogdt N., Krasniqi A., Lauwers Y., Zhao Y.J., D’Huyvetter M., Dumoulin M. (2021). A Non-Internalised CD38-Binding Radiolabelled Single-Domain Antibody Fragment to Monitor and Treat Multiple Myeloma. J. Hematol. Oncol..

[114] Quelven I., Monteil J., Sage M., Saidi A., Mounier J., Bayout A., Garrier J., Cogne M., Durand-Panteix S. (2020). 212Pb α-Radioimmunotherapy Targeting CD38 in Multiple Myeloma: A Preclinical Study. J. Nucl. Med..

[115] Teiluf K., Seidl C., Blechert B., Gaertner F.C., Gilbertz K.-P., Fernandez V., Bassermann F., Endell J., Boxhammer R., Leclair S. (2015). α-Radioimmunotherapy with ^213^Bi-Anti-CD38 Immunoconjugates Is Effective in a Mouse Model of Human Multiple Myeloma. Oncotarget.

[116] O’Steen S., Comstock M.L., Orozco J.J., Hamlin D.K., Wilbur D.S., Jones J.C., Kenoyer A., Nartea M.E., Lin Y., Miller B.W. (2019). The α-Emitter Astatine-211 Targeted to CD38 Can Eradicate Multiple Myeloma in a Disseminated Disease Model. Blood.

[117] Minnix M., Adhikarla V., Caserta E., Poku E., Rockne R., Shively J.E., Pichiorri F. (2021). Comparison of CD38-Targeted α- Versus β-Radionuclide Therapy of Disseminated Multiple Myeloma in an Animal Model. J. Nucl. Med..

[118] Sammartano V., Franceschini M., Fredducci S., Caroni F., Ciofini S., Pacelli P., Bocchia M., Gozzetti A. (2023). Anti-BCMA Novel Therapies for Multiple Myeloma. Cancer Drug Resist..

[119] Xing L., Liu Y., Liu J. (2023). Targeting BCMA in Multiple Myeloma: Advances in Antibody-Drug Conjugate Therapy. Cancers.

[120] Guo R., Lu W., Zhang Y., Cao X., Jin X., Zhao M. (2022). Targeting BCMA to Treat Multiple Myeloma: Updates From the 2021 ASH Annual Meeting. Front. Immunol..

[121] Swan D., Madduri D., Hocking J. (2024). CAR-T Cell Therapy in Multiple Myeloma: Current Status and Future Challenges. Blood Cancer J..

[122] Ma J., Zhang S., Yang N., Shang J., Gao X., Chen J., Wei H., Li Y., Zeng H., Xu H. (2025). Discovery of a Highly Specific Radiolabeled Antibody Targeting B-Cell Maturation Antigen: Applications in PET Imaging of Multiple Myeloma. Eur. J. Nucl. Med. Mol. Imaging.

[123] Comstock M.L., O’Steen S., Lin Y., Hamlin D., Wilbur D.S., Orozco J.J., Storb R.F., Walter R.B., Ataca Atilla P., Till B.G. (2023). BCMA-Directed Low Dose Alpha-Emitter Therapy Eliminates Minimal Residual Disease in a Multiple Myeloma Mouse Xenograft Model. Blood.

[124] Malaer J.D., Mathew P.A. (2017). CS1 (SLAMF7, CD319) Is an Effective Immunotherapeutic Target for Multiple Myeloma. Am. J. Cancer Res..

[125] Gormley N.J., Ko C.-W., Deisseroth A., Nie L., Kaminskas E., Kormanik N., Goldberg K.B., Farrell A.T., Pazdur R. (2017). FDA Drug Approval: Elotuzumab in Combination with Lenalidomide and Dexamethasone for the Treatment of Relapsed or Refractory Multiple Myeloma. Clin. Cancer Res..

[126] Ghai A., Zheleznyak A., Mixdorf M., O’Neal J., Ritchey J., Rettig M., DiPersio J., Shokeen M., Achilefu S. (2021). Development of [89Zr]DFO-Elotuzumab for immunoPET Imaging of CS1 in Multiple Myeloma. Eur. J. Nucl. Med. Mol. Imaging.

[127] De Veirman K., Puttemans J., Krasniqi A., Ertveldt T., Hanssens H., Romao E., Hose D., Goyvaert C., Vlummens P., Muyldermans S. (2021). CS1-Specific Single-Domain Antibodies Labeled with Actinium-225 Prolong Survival and Increase CD8+ T Cells and PD-L1 Expression in Multiple Myeloma. Oncoimmunology.

[128] Flieswasser T., Van den Eynde A., Van Audenaerde J., De Waele J., Lardon F., Riether C., de Haard H., Smits E., Pauwels P., Jacobs J. (2022). The CD70-CD27 Axis in Oncology: The New Kids on the Block. J. Exp. Clin. Cancer Res..

[129] Sauer T., Parikh K., Sharma S., Omer B., Sedloev D., Chen Q., Angenendt L., Schliemann C., Schmitt M., Müller-Tidow C. (2021). CD70-Specific CAR T Cells Have Potent Activity against Acute Myeloid Leukemia without HSC Toxicity. Blood.

[130] Hagemann U.B., Mihaylova D., Uran S.R., Borrebaek J., Grant D., Bjerke R.M., Karlsson J., Cuthbertson A.S. (2017). Targeted Alpha Therapy Using a Novel CD70 Targeted Thorium-227 Conjugate in in Vitro and in Vivo Models of Renal Cell Carcinoma. Oncotarget.

[131] Sonmezoglu K., Vatankulu B., Elverdi T., Akyel R., Erkan M.E., Halac M., Ocak M., Demirci E., Aydin Y. (2017). The Role of 68Ga-DOTA-TATE PET/CT Scanning in the Evaluation of Patients with Multiple Myeloma: Preliminary Results. Nucl. Med. Commun..

[132] Uijen M.J.M., Derks Y.H.W., Merkx R.I.J., Schilham M.G.M., Roosen J., Privé B.M., van Lith S.a.M., van Herpen C.M.L., Gotthardt M., Heskamp S. (2021). PSMA Radioligand Therapy for Solid Tumors Other than Prostate Cancer: Background, Opportunities, Challenges, and First Clinical Reports. Eur. J. Nucl. Med. Mol. Imaging.

[133] Miceli A., Riondato M., D’Amico F., Donegani M.I., Piol N., Mora M., Spina B., Morbelli S., Bauckneht M. (2021). Concomitant Prostate Cancer and Hodgkin Lymphoma: A Differential Diagnosis Guided by a Combined 68Ga-PSMA-11 and 18F-FDG PET/CT Approach. Medicina.

[134] Kanthan G.L., Coyle L., Kneebone A., Schembri G.P., Hsiao E. (2016). Follicular Lymphoma Showing Avid Uptake on 68Ga PSMA-HBED-CC PET/CT. Clin. Nucl. Med..

[135] Wang Z., Cong Y., Shi L., Jiang Y., Zhang H. (2023). Multiple Myeloma with Diffuse Uptake on 18F-PSMA-1007 Positron Emission Tomography/Computed Tomography: A Case Description and Literature Review. Quant. Imaging Med. Surg..

[136] Yang Y., Vedvyas Y., Alcaina Y., Son J.Y., Min I.M., Jin M.M. (2024). Low-Dose Targeted Radionuclide Therapy Synergizes with CAR T Cells and Enhances Tumor Response. Front. Immunol..

